# Pre-movement changes in sensorimotor beta oscillations predict motor adaptation drive

**DOI:** 10.1038/s41598-020-74833-z

**Published:** 2020-10-21

**Authors:** Henry T. Darch, Nadia L. Cerminara, Iain D. Gilchrist, Richard Apps

**Affiliations:** 1 School of Physiology, Pharmacology and Neuroscience, University of Bristol, Bristol, BS8 1TD UK; 2 School of Psychological Science, University of Bristol, Bristol, BS8 1TU UK; 3 Present Address: APC Microbiome Ireland, University College Cork, Cork, Ireland

**Keywords:** Motor control, Motor cortex

## Abstract

Beta frequency oscillations in scalp electroencephalography (EEG) recordings over the primary motor cortex have been associated with the preparation and execution of voluntary movements. Here, we test whether changes in beta frequency are related to the preparation of adapted movements in human, and whether such effects generalise to other species (cat). Eleven healthy adult humans performed a joystick visuomotor adaptation task. Beta (15–25 Hz) scalp EEG signals recorded over the motor cortex during a pre-movement preparatory phase were, on average, significantly reduced in amplitude during early adaptation trials compared to baseline, late adaptation, or aftereffect trials. The changes in beta were not related to measurements of reaction time or reach duration. We also recorded local field potential (LFP) activity within the primary motor cortex of three cats during a prism visuomotor adaptation task. Analysis of these signals revealed similar reductions in motor cortical LFP beta frequencies during early adaptation. This effect was present when controlling for any influence of the reaction time and reach duration. Overall, the results are consistent with a reduction in pre-movement beta oscillations predicting an increase in adaptive drive in upcoming task performance when motor errors are largest in magnitude and the rate of adaptation is greatest.

## Introduction

Motor adaptation, the ability to modify movements in response to changes in the environment to maintain accuracy, is a fundamental feature of most types of goal-directed behaviour. Motor adaptation is supported by a supraspinal neural network that includes (but is not limited to) the cerebellum and motor cortex. These brain regions are thought to serve different, but complementary roles during motor adaptation. For example, a large body of work indicates the cerebellum is critically involved in the generation of sensory prediction errors that drive the adaptation process, as well as online control of complex movements^1–4^. In contrast, the motor cortex appears to support memory of different environment dynamics: Transcranial magnetic disruption of the motor cortex in humans does not influence learning of a new perturbing force, but leads to a deficit in behavioural performance compared with controls in a delayed re-test of the same force^5^, and non-invasive electrical stimulation of the motor cortex can improve retention of motor adaptation performance^6^. Taken together, these findings suggest that motor cortical areas support a long-term memory trace of established motor programs, into which newly learned dynamics (potentially computed initially in the cerebellum) are integrated.

Electroencephalography (EEG) signals in the beta frequency band are usually defined as being between 13 and 30 Hz^7,8^. They are a prominent feature of motor areas in both human and other mammalian species (including primates^9,10^, and cats^11,12^ cited in Ref.^13^). There is now a well-established link between beta frequencies and movement-related processing in the sensorimotor cortex^14^. Typically, beta frequency EEG responses in the motor cortex are reduced in amplitude during movement (event-related desynchronization)^15^ and increased at cessation of movements (beta rebound)^8^. Beta amplitude also increases during periods of sustained holding^16–18^ and has been linked to various kinematic parameters including reaction time^19^ and movement speed^20^.

In the context of motor adaptation, only a limited number of studies have examined motor cortical beta signals in peri-movement time periods. The beta rebound has been observed to be attenuated during trials in which large errors are made^21^, and this has been attributed to a reduction in the subject’s confidence in the motor program, prompting subsequent adjustment or exploratory movements^22^. This is considered to reflect a role in feedback error processing to modify subsequent motor actions, possibly indicating an *adaptive drive*—defined here as neuronal signals generated in response to an increase in behavioural error specifically computed to support modification of subsequent motor actions. Motor cortical beta signals have also been reported to be attenuated prior to movement initiation in both a forcefield and a visuomotor adaptation task^23^. This pre-movement reduction in beta power was interpreted to reflect a predictive updating of the upcoming motor command in response to a previous error, and thereby signalling an adaptive drive^23^. However, this idea was challenged by a subsequent study that used an interlimb cooperative adaptation task to decouple adaptive drive from altered motor commands and found that premovement beta modulation might be a result of higher-level processing of sensory information that updates upcoming movements^24^. The present study sought to clarify whether changes in pre-movement beta oscillations are consistent with an increase in adaptive drive.

The results outlined above relied on recording EEG signals obtained from scalp electrodes. The EEG signal of individual electrodes are thought to reflect a spatial sum of a large neuronal population subjacent to them^25,26^. It remains to be established whether any adaptation related changes in motor cortical signals present in scalp EEG are also present in neural population activity recorded directly from the brain as a local field potential (LFP). LFP recordings from the motor cortex are not available in healthy human participants during motor adaptation, so in addition to recording EEG signals over the motor cortex in healthy humans, we also recorded LFP directly from the motor cortex in an animal model of visuomotor adaptation, using a previously established reach-retrieve task in cats. This allowed us to test whether changes in beta activity in motor cortex could be related to adaptive drive, and whether such changes are generalizable across species. We focus on beta activity in the pre-movement period when adaptive drive is greatest for upcoming task performance. This pre-movement period has the advantage that it is not confounded by the execution of the movement (which differs across the motor adaptation tasks in the two species), allowing a direct comparison of results between the two lines of experiment.

We found that beta frequency power recorded over the motor cortex as scalp EEG in humans, or directly from the primary motor cortex as LFP activity in cats, was reduced prior to movement in trials in the early adaptation phase of a joystick visuomotor adaptation task (humans) or when target mis-reaches occurred in a prism visuomotor adaptation task (cats). These changes in beta power were not related to kinematics of the reach, consistent with a role of reduced beta activity in the motor cortex reflecting adaptive drive. Interestingly, in both cases the effect was restricted to early adaptation, but not the early phase of the aftereffect (in which targeting errors also occur), suggesting a dissociation in neuronal activity between adaptation and washout periods.

## Methods

### Ethics

Animal experiments were carried out in accordance with the UK Animals (Scientific Procedures) Act 1986 regulation and were reviewed and approved by the University of Bristol Animal Welfare Ethical Review Body. The human experiments were approved by the University of Bristol Faculty of Science Ethics Committee. All procedures were carried out in accordance with the approved guidelines and. participants provided written informed consent prior to participation. Participants were remunerated for their time in accordance with local policy.

### Behaviour

#### Human experiments

Eleven healthy young adults took part (aged between 21 and 31, 4 female). All were right-handed (Edinburgh Handedness Inventory score: + 87.5, SD ± 14.3) and had normal or corrected to normal vision.

**Figure 1:**
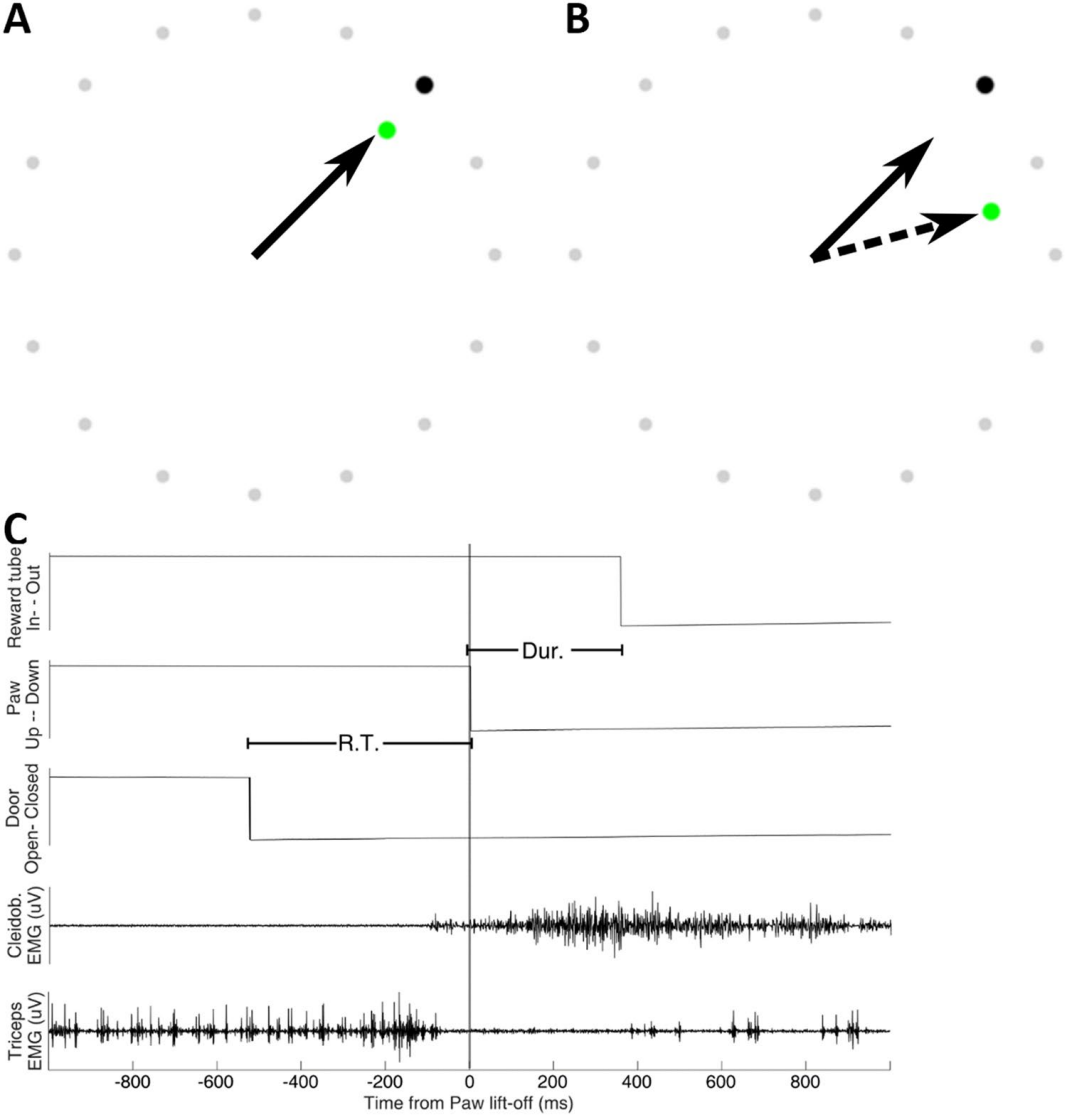
Example view of human reaching experiment; grey circles indicate the task arena boundary which participants were instructed to move the joystick through. (**A**) During baseline and aftereffect blocks, the green cursor follows the trajectory of the joystick (arrow). (**B**) To induce visuomotor adaptation during the Adaptation block, a 30^o^ clockwise rotation to the position of the green cursor was implemented, providing the illusion of an incorrect joystick trajectory (dashed arrow). (**C**) Example event markers and muscle activity recorded during the cat forelimb reaching movement in one trial. Tonic bursting activity of triceps (extensor) muscles ceases immediately before the forelimb breaks contact with the contact plate. At this time, Cleidobrachialis (flexor) muscle begins a ramped increase in activity that lasts for the duration of the reach, with activity continuing beyond as the animal collects the food reward. R.T., reaction time in response to cue to start reach; Dur, duration of reaching movement to target.

Participants performed a visuomotor adaptation task requiring them to use a joystick to control an on-screen cursor^21,27^. Each trial was initiated by the participant depressing the joystick trigger. One of four possible black target dots (45°, 135°, 225°, or 315° from vertical) immediately appeared at the edge of an invisible boundary circle centred on the starting position of the user-controlled green dot, forming the task arena (Fig. 1A,B). Targets were pseudorandomly presented in blocks of 20 trials, with each target location presented 5 times within each block. Participants were instructed to move the joystick as quickly and accurately as possible to the target, so the movements were effectively ballistic. Trials that exceeded 750 ms between target presentation and completion elicited an on-screen prompt to speed up the movements. The joystick was positioned in front of the dominant (right) hand to the right-hand side of the screen, enabling a comfortable grasp whilst removing the hand from the participant’s view whilst engaged in the task. The task was run with Matlab using Psychtoolbox version 3. Task code was modified from that made publicly available by D. Goldschmidt at https://github.com/degoldschmidt/motor-experiments. At the end of each individual trial, the green cursor froze at the point of crossing the boundary of the task space until the participant began the next trial, delivering a stable accuracy marker after the rapid movement.

In the human study there were three trial blocks each of 200 individual reach trials. The first block consisted of ‘baseline’ trials in which the participants performed the joystick task as normal. The second block induced visuomotor adaptation by implementing a clockwise 35° rotation transformation of the on-screen joystick (green) cursor, requiring participants to modify their outward movement to overcome this perturbation. The final block, identical to the first, was used to probe the participants’ behavioural adaptation to the transformation through the presence of an aftereffect. Participants were not informed of the rotation transformation or the ordering of each block, and none reported explicit awareness of the rotation transform other than that their performance had worsened. Participants did not report the use of an explicit aiming strategy.

Measurements of behavioural error were calculated as the angular displacement relative to the target when reaches crossed the boundary of the task space (endpoint error)^28,29^. Movement initiation was determined when the joystick cursor exceeded a Euclidian distance of 5 pixels from the starting position. Reaction time was measured between the time of target presentation (joystick trigger press), and movement initiation. Reach duration was measured between the time of movement initiation and crossing of the task boundary.

#### Animal experiments

Three adult male cats (B, S and G) were trained to perform a left forelimb reach-retrieve task. The target was a Perspex tube (diameter 3 cm) placed at eye level at a comfortable reaching height and distance (approximately 20 cm) in front of the animal^30^. Reaches were cued by an opaque vertical covering on the Perspex tube which was operated manually by the researchers when the cat was attending to the door and had the reaching limb resting on the ground. The time taken between the door opening and the paw lift-off was considered the reaction time. The reach duration was also recorded as the time between the paw lift-off and entry into the tube, detected by infra-red beams. Reaches were always positively rewarded with a fish morsel within the Perspex tube and no negative training or food restriction was used. Video recordings of the behaviour were made using webcams running at 30 fps, synchronised with the neural data acquisition using the open source software Bonsai^31^. These videos were assessed offline to classify reaches (see below).

Once the animals had learnt the reaching task (typically 4 weeks training) surgical implants of recording electrodes were made (see below). Following full recovery from the surgery, daily recording sessions commenced in which the cats performed approximately 80 food-rewarded reaching trials before they became sated and the session was stopped, and they were returned to their home pen. The progression of each recording session followed that of the human study and was divided into three stages. Stage 1: twenty baseline trials were obtained in which unperturbed reaching was performed and animals rapidly and smoothly placed their forepaw into the target reward tube. Stage 2: visuomotor adaptation trials in which a set of custom made 40 dioptre Fresnel laterally displacing prism glasses were placed in the animal’s line of sight (approximately 2 cm in front of the eyes). Initial trials in the presence of the prism glasses led to reaches laterally missing the entrance of the target reward tube. Adaptation to successfully reach into the target occurred after approximately 10 trials. After 20 consecutive accurate reaches, the prism glasses were removed. Stage 3: immediately after removal of the prism glasses the cats exhibited lateral misses to the opposite side of the tube, indicative of a motor adaptation aftereffect.

Based on experimenter observation during the recording session and offline verification by examining a video record of task performance, individual reaches were divided into three categories: (i) ‘Hits’ when the cat rapidly and smoothly reached to place its forepaw into the target reward tube. (ii) ‘Misses’ were the same as hits for the reach component of the movement, but the forepaw failed initially to enter the target, hitting the Perspex façade ~ 1 cm to the left or right of the reward tube entrance (ie. opposite to the original prism glasses displacement). A subsequent lateral adjustment was required for the paw to enter the tube to retrieve the reward. (iii) ‘Rejected’ trials were all reaches that either had a reaction time shorter than 150 ms; a reach duration (time between paw lift-off and target/façade hit) more than 2 standard deviations of the mean computed for each animal; reaches that displayed a corrective adjustment near the end of the movement, prior to contact with the Perspex façade, in order for the forepaw to enter the reward tube; or trials with large electrical noise artefacts in the neural data. Rejected reaches accounted for approximately 20% of all trials and are not considered further.

#### Trial epochs

For the human experiments, data from five epochs, each of 72 consecutive trials, was extracted from each participant. The five epochs were: baseline—the final trials of the baseline period; early adaptation—the set of trials following the visuomotor rotation, in which endpoint errors were greater than 2SD from baseline; late adaptation—final trials under the visuomotor rotation condition; early aftereffect—the 72 trials following the removal of the visuomotor rotation, in which endpoint errors were greater than 2SD from Baseline; and late aftereffect—the final 72 trials of the task.

For the cat behaviour, ‘Hits’ made prior to the prism perturbation were taken as equivalent to the baseline condition in the human experiments. The ‘Miss’ category is taken as equivalent to the Early Adaptation period when errors are largest in magnitude and the rate of adaptation is greatest, while ‘Hits’ made during prism perturbation (when adaptation had occurred) were taken as equivalent to the human Late Adaptation trials. Analysis of aftereffect trials was not possible in the animal experiments because of the limited number of available trials.

### Electrophysiological recordings

#### Human EEG

The experiments were preformed within a purpose-built Faraday cage. EEG signals were captured using 32 active electrodes (ActiCAP, BrainProducts) arranged according to the extended international 10/20 system. Electrode preparation proceeded as per the manufacturers guidance and impedances were reduced to < 5 kΩ with conductive gel (SuperVisc, BrainProducts). Data were recorded at 2500 Hz using a BrainAmp DC amplifier and BrainVision Recorder Software (BrainProducts).

#### Animal LFP

General surgical methods have been reported elsewhere^30^. Briefly, anaesthesia was induced with Propofol (i.v. 10 mg/kg, Abbott Animal Health) and maintained with gaseous isofluorane. A veterinary anaesthetist was present to administer anaesthetic agents and monitor vital signs. Stereotaxic surgery involved a craniotomy over the left motor cortex (approximate coordinates: AP + 23, ML + 8). An area corresponding to the distal left forelimb motor cortex was identified through microstimulation (11 pulse 330 Hz trains^32^) of the cortical surface around the cruciate sulcus. Next, a 16-site silicon probe (NeuroNexus) was implanted into the identified area. The probe consisted of sixteen 2 mm shanks, spaced 100 µm apart, with a single recording site at each tip. Sites were referenced to a skull screw situated contralateral to the craniotomy. Additionally, EMG recording leads (multistranded stainless steel, Teflon insulated, diameter 0.3 mm, Cooner, Chatsworth, CA, USA) were implanted into the left forelimb Cleidobrachialis Flexor, as well as the long and lateral heads of Triceps Brachii Extensor muscles. A Teflon insulated lead was also implanted into connective tissue in the left forepaw wrist. This lead was used to carry a 400 mV 30 kHz sine wave transmitted through the paw when in contact with a copper baseplate on the floor of the recording environment, enabling the detection of paw lift-off at the initiation of each reach. Animals recovered from surgery in the home pen for 1 week prior to commencement of daily recording sessions (see above).

Data from the neural probe were captured at 30 kHz with a Cerberus neural acquisition processor (Blackrock Microsystems). EMG signals were online filtered with analog filters (bandpass 0.3–5 kHz) and visualised on a digital oscilloscope and then recorded at 30 kHz by the Cerberus processor. The paw contact signal was converted into an analog TTL signal to indicate paw contact with the base plate and time of paw lift. Paw entry into the tube was determined by a photo-electric switch at the tube entrance.

### Data pre-processing

#### Human EEG

EEG data were offline pre-processed using EEGLab^33^. For each participant, data were decomposed using the Infomax Independent Component Analysis Algorithm, with components representing noise artefacts (such as eye blinks or neck musculature) identified and removed before back-transforming the data into time-domain electrode signals^34,35^. Based on extensive prior work attributing movement related beta activity to the motor cortex^8,36^ and to better isolate signals originating from the motor cortex, a Hjorth approximation of the surface Laplacian^37^ using the 4 nearest neighbour electrodes at C3 and C4 electrodes was computed to represent signals originating from contra-/ipsilateral motor cortex respectively^38–42^. Trials were identified from the timestamps of the joystick trigger press (target presentation), and movement initiation was detected when the Euclidian distance of the cursor extended beyond a predetermined ‘zero’ zone (5 pixels).

#### Animal LFP

Data captured during individual recording sessions were visually inspected in Spike2 (Cambridge Electronic Design) for recording quality and timestamps for reach trials were identified and extracted based on the paw contact signal. LFP recordings across the 16 motor cortical sites were highly similar. Consequently, an example channel was selected, and re-referenced to a second channel at the opposite end of the probe (reference site spacing 1.1 mm). This removed any low frequency artefact due to movement and provided a measure of local oscillatory activity within the brain.

### Data analysis

Further data analysis was performed using custom made Matlab functions. Pre-processed data from both human and animal experiments were low-pass (30 Hz) filtered, down-sampled to 500 Hz and high-pass (10 Hz) filtered (4th order Butterworth). Next, 2 s windows centred on the movement initiation (joystick movement or paw lift-off event marker) were extracted, and z-score normalised. The time-series were then decomposed into the time–frequency domain using a Morlet wavelet transform (k = 6)^43^, and spectral power computed by taking the log normalised squared magnitude of the wavelet transform.

Based on our interest in motor preparation, we computed the mean spectral power during the reaction time between target presentation and movement initiation. In the case of the cats, as we had access to electromyographic (EMG) data from the Cleidobrachialis Flexor, responsible for lifting the forelimb, we were able to determine a suitable location for the window from the trial averaged EMG traces. The flexor muscle is consistently quiescent when the animal is sitting quietly but has a robust ramping activity during reaching that onsets prior to the timestamp of the paw lift (Fig. 1C). Thus, a 200 ms window was positioned immediately prior to this ramping activity (Cats B and G: 400–200 ms prior to paw lift. Cat S: 100–300 ms prior to paw lift). As we did not have access to the equivalent EMG data for human participants, the full reaction time period was used.

### Experimental design and statistical analyses

These experiments are formally exploratory as prior to the experiment there was no suitable data to carry out an a-priori power calculation. In addition, ethical issues restricted the number of animals in the study. All statistical analyses were performed in SPSS (IBM).

#### Human data

Data from all participants were included in a General Linear Mixed-Model (GLMM) to assess the effect of adaptation condition, with Bonferroni corrected pairwise post-hoc comparisons. Participant ID was used as a between subjects factor. Trial number and adaptation condition were considered repeated effects. Residuals were inspected for near-normality with P–P plots.

#### Animal data

Each animal was analysed as an individual case study, and statistical analyses performed with bootstrapped data (n = 1000) in order to balance comparison groups. Data from each animal were subject to a Univariate ANOVA assessing the effects of adaptation condition. Reaction times and durations were incorporated as covariates. Post-hoc pairwise comparisons were made using Bonferroni correction. For all tests, data were checked for normality and homogeneity of variance where appropriate.

## Results

**Figure 2:**
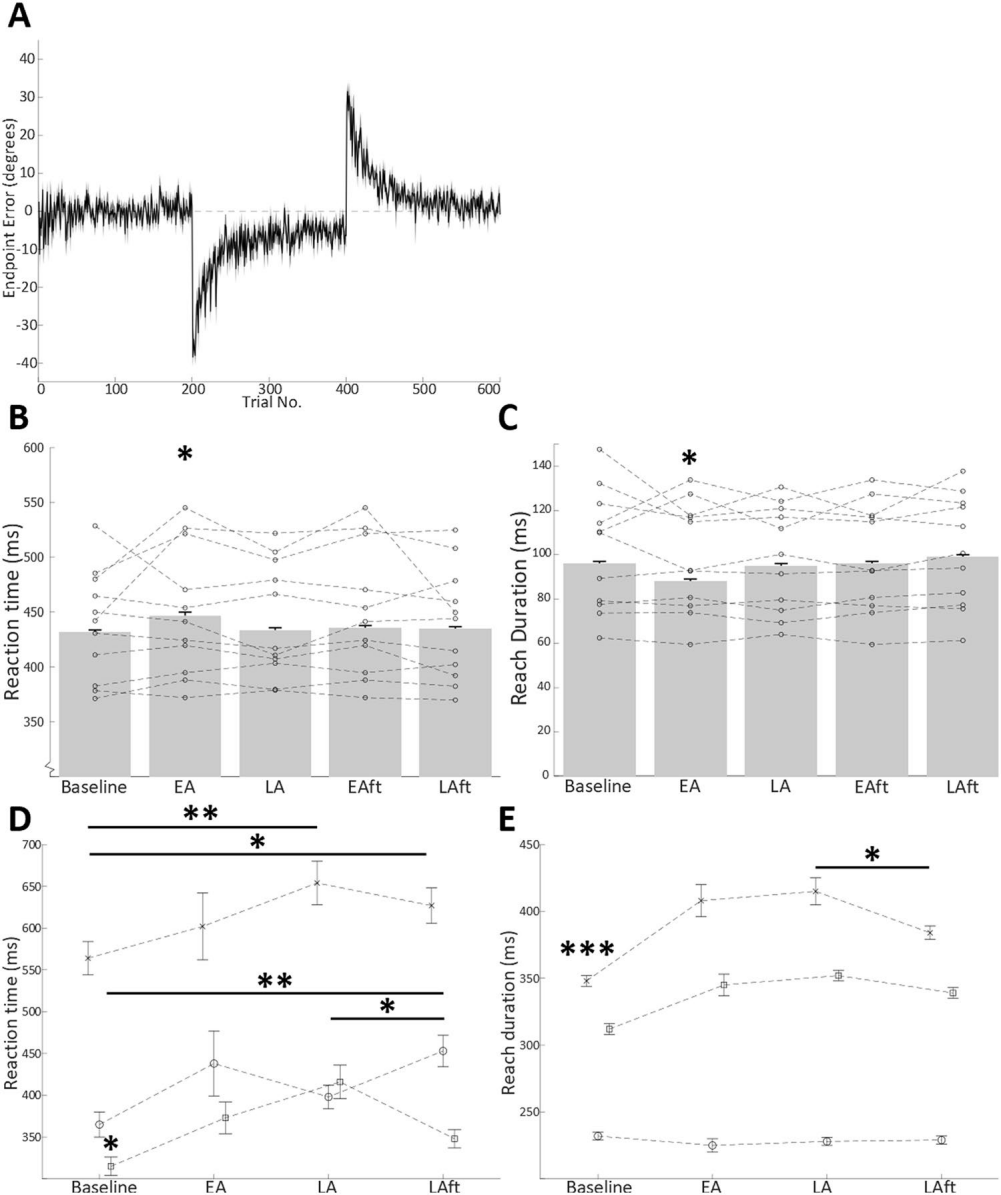
(**A**) Human task grand average trial-by-trial endpoint-error. Negative error indicates a clockwise error direction. Shaded area represents standard error. During the first 200 trials (including Baseline epoch), endpoint errors are near zero. When the 30° clockwise perturbation is applied on trials 201–400, motor adaptation occurs as shown by a progressive reduction in endpoint errors until asymptote is reached at around 5°–7° of clockwise error. When the 30° rotation is removed in the final 200 trials, a negative aftereffect occurs, with an initial error opposing that of initial adaptation, returning to baseline performance levels by the final trials. Bar charts represent grand average reaction time (**B**) and reach duration (**C**) during adaptation epochs of human task. (**D**, **E**) Mean reaction time and reach duration over adaptation epochs in cats (**A**) (cross mark), (**B**) (circle), and (**C**) (square). Error bars indicate standard error. Individual data points represent participant means. Asterisks indicate Bonferroni corrected pairwise comparison to other groups, *p < 0.05, **p < 0.01). *Baseline* baseline epoch, *EA* early adaptation epoch, *LA* late adaptation epoch, *EAft* early aftereffect epoch, *LAft* late aftereffect epoch.

**Figure 3:**
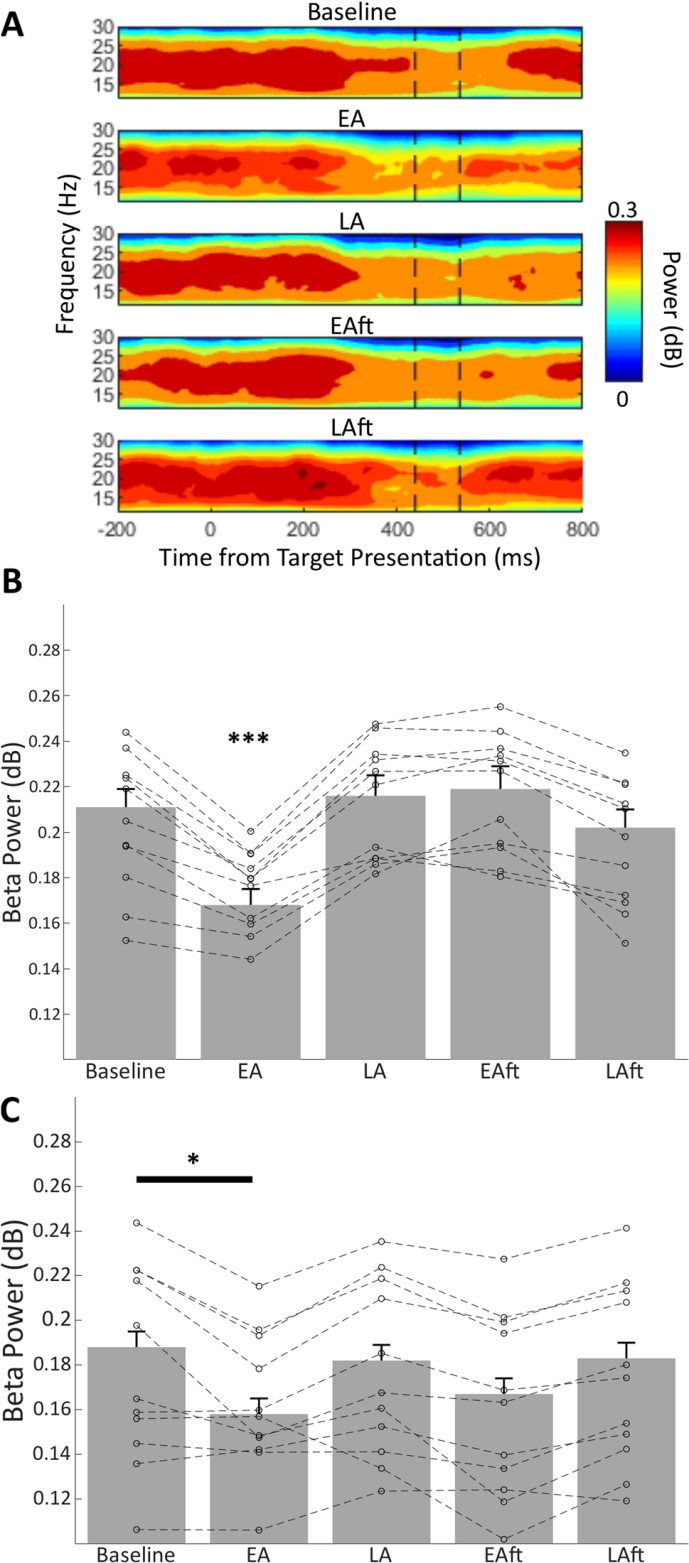
Grand mean spectrogram of human participant contralateral beta signal, aligned to target presentation at time zero (**A**). Vertical dashed black lines from left to right denote mean reaction time and end of reach respectively. Bar charts indicate group mean beta power in each adaptation epoch for contralateral (**B**) and ipsilateral (**C**) motor cortex, controlling for effects of reaction time and reach duration. Datapoints linked by dashed lines represent individual participant means. ***Bonferroni corrected pairwise significance (p < 0.0005) to each other epoch. *Bonferroni corrected pairwise significance (p < 0.05) for comparison to baseline epoch. *Baseline* baseline epoch, *EA* early adaptation epoch, *LA* late adaptation epoch, *EAft* early aftereffect epoch, *LAft* late aftereffect epoch.

In the human joystick task, the un-cued introduction, and later removal, of a 30° rotation to visual feedback of endpoint performance led to classical motor adaptation behaviour (Fig. 2A). Specifically, after an initial perceived endpoint error of 38.45° ± 1.1, there was a rapid decay of endpoint error until an asymptote of around 5.38° ± 0.83. After the removal of the rotation, the participants exhibited a negative aftereffect (− 27.84° ± 0.86), which rapidly decayed to near baseline performance (final 10 trials mean: − 0.85° ± 0.55). In line with previous studies we observed a movement related suppression of beta activity 200–300 ms after target presentation that persisted beyond the end of the outbound reach (Fig. 3A).

### Pre-movement beta does not predict endpoint error magnitude

An intrinsic feature of adaptive behaviour is the changes in behavioural success. Previously, post-movement beta signals have been linked to the assessment of the accuracy of the previous trial^22^ . As we had access to the endpoint errors in the human task (but not in the cats), we were able to assess whether pre-movement beta signals also carry information regarding reach accuracy. Regression of the trial-by-trial pre-movement beta power and the subsequent endpoint error magnitude found no significant effect in either contralateral: F(1,593) = 2.55, p = 0.111; or ipsilateral: F(1,602) = 1.95, p = 0.163; signals. Therefore, changes to the pre-movement beta power do not scale with the experienced behavioural errors.

### Pre-movement beta predicts reaction time and reach duration

Previous literature in multiple species provides evidence that beta signals in the sensorimotor cortex may contain information about the timing and kinematics of voluntary movements^19,44,45^, although this is not always the case^46^. To determine whether such interactions were present in pre-movement beta signals, we correlated the trial-by-trial beta data to the measured reaction time and reach duration of the upcoming reach. In both species, we found a significant non-parametric correlation between pre-movement beta power and both metrics (Human: ρ_ReactionTime_ = 0.053, p = 0.002, ρ_ReachDuration_ = − 0.103, p < 0.0005; cat: ρ_ReactionTime_ = − 0.76, p = 0.002, ρ_ReachDuration_ = − 0.537, p < 0.0005).

Furthermore, we found a significant change in these behavioural measures during Early Adaptation in humans (Fig. 2B,C), with a 15 ms slower reaction time, but an 8 ms shorter reach duration relative to baseline, presumably as the participants sped up the reaches to maintain an overall trial duration within the allowed 0.75 s (F_ReactionTime_(5,525) = 31,424 , p < 0.0005, F_ReachDuration_ (5,557) = 8964 , p < 0.0005).

These measures also significantly changed throughout the task in the three cats (Fig. 2D,E). Broadly, both reaction time, and reach duration was shortest during baseline trials and greatest in Late Adaptation trials.

These results therefore suggest that pre-movement beta signals in the motor cortex may contain information relating to the kinematics of the upcoming reach, and that movement parameters are not uniform over the different stages of motor adaptation. To take this into account we therefore incorporated both metrics as covariates in subsequent analyses of the dynamics of beta power through the adaptation epochs.

### Bilateral pre-movement EEG beta power is suppressed in early adaptation, but not during aftereffect

The primary aim of this study was to assess whether the pre-movement beta signals in the motor cortex contained information relating to adaptive processes, that could indicate enhanced sensorimotor processing and adaptive drive. In the human EEG data, GLMM analysis revealed a significant main effect of adaptation condition for both contralateral (F(5,893) = 31.57, p < 0.0005, Fig. 3B) and ipsilateral hemispheres (F(5,743) = 67.79, p < 0.0005, Fig. 3C). Post-hoc comparisons revealed that Early Adaptation trials had significantly lower contralateral beta power compared to all other epochs (p ≤ 0.013), while ipsilateral beta was only significantly decreased compared to the baseline epoch (p = 0.031).

### Pre-movement motor cortical LFP beta power is suppressed in early adaptation

**Figure 4:**
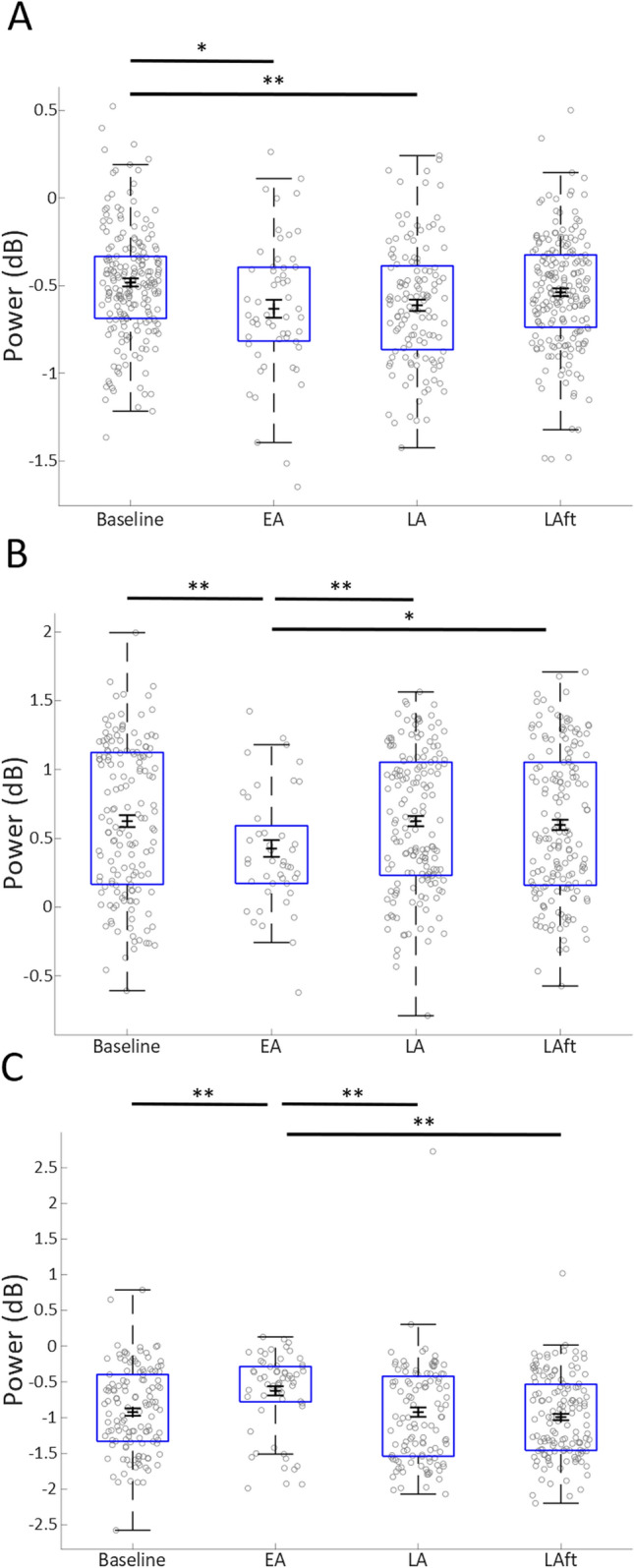
Pre-movement Beta LFP power in three cats (**A**–**C**). Box-whisker plot showing distribution of analysed data (individual trials shown in grey circles), in each major adaptation epoch considered. Black error bars show mean and standard error of bootstrap re-sampling (n = 1000) of data. Asterisks show significant pairwise comparisons (Bonferroni corrected), *p < 0.05, **p < 0.01. *Baseline* baseline epoch, *EA* early adaptation epoch, *LA* late adaptation epoch, *EAft* early aftereffect epoch, *LAft* late aftereffect epoch.

Analyses of the motor cortical LFP signals in two of the three adult male cats revealed a similar adaptation related effect to that observed in the human data (i.e. decreased beta power in Early Adaptation), with an opposing effect in cat C (Fig. 4). In all three animals, a bootstrapped (n = 1000) General Linear Model, including reaction time and reach duration as covariates, found a significant effect of adaptation epoch (cat A: F(4,590) = 24.162, p < 0.0005. Cat B: F(4,528) = 11.380, p < 0.0005. Cat C: F(4,443) = 6.577, p < 0.0005). Bonferroni post-hoc comparisons confirmed that the baseline-Early adaptation change in all three animals was significant (cat A; p = 0.011, cat B; p = 0.006, cat C; p = 0.001), and the Early adaptation-Late adaptation changes were also significant for cats B and C (cat A; p = 0.749, cat B; p = 0.007, p = 0.001).

In summary, the averaged human EEG data show a reduction in beta power pre-movement in Early Adaptation compared to all other epochs. In two cats motor cortical LFP exhibited a similar pattern of modulation to the human data, with a pre-movement decrease in Early Adaptation compared to baseline and Late Adaptation, whilst a third animal showed the opposite effect. The inclusion of reaction time and reach duration as covariates in the analysis suggests that the adaptation specific modulation of beta is not related to these reach kinematics, and the lack of significant modulation of EEG data during the Early Aftereffect epoch also suggests that the beta modulation is not related to error feedback signals.

## Discussion

In this study we show that EEG beta frequencies in humans, recorded at a scalp location classically associated with motor cortical signals, are attenuated immediately prior to movement initiation specifically during early stages of visuomotor adaptation, and returned to baseline levels when behavioural performance (endpoint errors) had returned to baseline performance: i.e. when adaptative drive is greatest. Further, we provide evidence that beta activity recorded as local field potential activity directly from the motor cortex in cats exhibits similar motor adaptation related changes.

Our results are consistent with previous work in human subjects which show a decrease in pre-movement motor cortical EEG beta power during early stages of forcefield, and visuomotor adaptation task—involving perturbing forces being applied to the limb^23,24^. Forcefield adaptation normally involves the integration of visual and proprioceptive information^47^, whereas visuomotor adaptation relies on visual feedback to monitor performance^48^. This would suggest the change in beta activity within the motor cortex is independent of the mode of sensory feedback. However, previous studies indicate that forcefield and visuomotor adaptation may involve different neural pathways/processes within the cerebellar cortex^49–52^. One possible explanation to unite these findings is that signals arising from distinct cerebellar cortical regions are integrated within the cerebellar nuclei and/or thalamus before being forwarded to the motor cortex for the preparation of subsequent limb movements.

Our data are also consistent with the finding that ipsilateral beta power is reduced prior to target presentation when participants expect a change in required motor program during a go/no-go task^53^. This reinforces the possibility that this phenomenon is not related to kinematic features of the motor programs themselves, but instead indicate an increased drive of adaptive mechanisms promoting motor flexibility.

Our results from three cats performing a prism visuomotor adaptation task provide additional evidence that a similar pre-movement modulation in beta power can also occur in motor cortical LFP signals. Individual differences observed within the animal data may indicate differences in neural population activity at a more local scale due to topographical differences in electrode positioning in relation to motor cortical maps. Nevertheless, two out of the three cats displayed the same adaptation related modulation of pre-movement beta power, suggesting that the effects are generalizable across species, and that the effects seen in the human EEG data are at least in part driven by motor cortical activity.

Reaction time and reach duration have previously been associated with beta oscillations in both humans and primates^19,20,54^. In our human data, the bilateral reduction in pre-movement beta activity in Early Adaptation trials was present when controlling for these parameters, suggesting that the change in beta activity is independent of these kinematic properties. Moreover, in the human data there was no significant change in beta power during the Early Aftereffect epoch, in which motor errors are equal in magnitude to the Early Adaptation epoch. This suggests that the beta modulation was not related to error feedback processing per se, which is thought to occur largely in the cerebellum^55–58^. Instead, the beta modulation was present only when adaptation occurred to a *novel* environment dynamic, indicating a more categorical indicator that adaptation of the motor programs are required (perhaps similar in nature to the decreased beta power when expecting a rule change in a Go/No-Go task^53^). The difference in beta activity between the Early Adaptation and Early Aftereffect epochs raises the possibility that the neuronal processes underpinning adaptation and aftereffect are distinct, and that the adaptation effect is not related to error magnitude. Such a possibility is consistent with the idea that, whilst initial adaptation is supported principally by a recalibration of internal models, the aftereffect can be more easily supported with a strategic influence^59^.

### Neural circuit basis of beta modulation

Simultaneous EEG, LFP and multi-unit spiking activity recording in area F5 (ventral pre-motor cortex) of macaques has previously shown an inverse relationship between multi-unit activity and LFP and EEG beta power^60^. Therefore, the present pre-movement beta modulation could reflect an increase in motor cortical spiking activity at times of increased adaptive drive. Indeed, beta oscillations have been linked with antidromic activity within pyramidal tract neurons of the motor cortex^61^, as well as the balance of inhibition/excitation (with elevated levels of endogenous GABA enhancing both baseline beta oscillations and the magnitude of the movement-related desynchronisation)^62–64^. Furthermore, studies of non-human primates during motor tasks has revealed that many cortical neurons synchronise their activity to beta rhythms during periods of oscillation, limiting their spiking rate to a narrower range than that present outside of oscillatory episodes^65^. Together, this raises the possibility that the decreased pre-movement beta power observed here and in previous studies reflects altered inhibitory control during times of increased adaptive drive.

An alternative (but not mutually exclusive) view of the origins of LFP oscillations is that they reflect synchronous activity of synaptic inputs to neurons local to the recording site^25,66,67^, occurring in bursts of one beta cycle^68^. The observed modulation of beta power could then be interpreted to represent changes in the amount of neural synchrony in afferent signals to the motor cortex as proposed by Torrecillos and colleagues^23^.

In conclusion, our results show an adaptation related attenuation of beta oscillations during movement preparation, recorded by scalp EEG over the motor cortex in humans and as motor cortical LFP signals in cats. Our findings are consistent with the reduction in beta power being a result of increased afferent input to the motor cortical PTNs in the early stages of adaptation, which could correspond to changes in the excitatory/inhibitory balance disrupting the ongoing beta oscillations, thus enabling modifications to established motor programs in response to error signals detected by other brain regions such as the cerebellum. Further work is necessary to elucidate the precise mechanisms supporting this beta phenomenon.

## Data Availability

The data included in this report are available from the corresponding authors upon reasonable request.
